# Omalizumab for severe IgE-mediated food allergy and severe asthma after pediatric liver transplantation: a case report

**DOI:** 10.3389/fped.2026.1855822

**Published:** 2026-06-17

**Authors:** Antonella Mosca, Andrea Ficari, Maria Sole Basso, Carmen Mazzuca, Daniela Liccardo, Alessandro Fiocchi, Andrea Pietrobattista, Vincenzo Fierro

**Affiliations:** 1 Hepatology and Liver Transplant Unit, Bambino Gesù Children’s Hospital, IRCCS, Rome, Italy; 2 Academic Department of Pediatrics, Bambino Gesù Children’s Hospital, IRCCS, Rome, Italy; 3 Division of Allergy, University Department of Pediatrics, Bambino Gesù Children’s Hospital, IRCCS, Rome, Italy

**Keywords:** *de novo* post-transplant food allergy, omalizumab, pediatric liver transplantation, severe asthma, severe food allergy

## Abstract

Transplant-associated allergy represents a clinically relevant phenomenon described after hematopoietic stem cell transplantation and, particularly in pediatric patients, after solid organ transplantation. Liver transplant (LT) in the pediatric population is associated with *de novo* IgE-mediated allergy, including asthma, atopic dermatitis, and severe food allergies. *De novo* post-transplant food allergy (dnPTFA) is a condition with multiple risk factors involved, including young recipient age, female sex, personal or donor history of atopy, calcineurin inhibitors, and Epstein–Barr virus infection. These factors likely converge to promote post-transplant immune dysregulation through both passive and active mechanisms driven by calcineurin inhibitor (CNI)-induced Th2 skewing and viral immune modulation. This paper presents a narrative review of dnPTFA and a clinical case of a 15-year-old boy who underwent LT at 28 days of age due to neonatal acute liver failure. The patient subsequently developed severe asthma and multiple dnPTFAs with recurrent anaphylaxis, despite immunosuppression being maintained at low target levels throughout post-transplant follow-up due to normal graft function. Because of persistent life-threatening reactions, allergic asthma, and marked food selectivity compromising growth, off-label treatment with omalizumab was initiated at 10 years of age. Following treatment with omalizumab, the patient achieved excellent asthma control, discontinued background therapy, and resumed a less restricted diet. Liver graft function has remained stable, with no graft-related adverse events over more than 5 years of follow-up, supporting the long-term safety and effectiveness of this approach.

## Introduction

Allergy is defined as an immune-mediated hypersensitivity reaction in which the immune system responds exaggeratedly or inappropriately to substances that are typically harmless, known as allergens. This response can be IgE-mediated (Type I hypersensitivity) but also includes non-IgE-mediated mechanisms and mixed forms. Transplant-associated allergy (TAA) has been reported in the literature in both pediatric and adult patients undergoing solid or non-solid organ transplantation, particularly in liver transplant (LT) recipients. *De novo* post-transplant food allergy (dnPTFA) is a relatively common phenomenon among pediatric transplant recipients, with reported prevalence ranging from 4% to 58%, significantly higher than the 0.45%–10% observed in the general population (1–3). Inhalant allergy-related diseases include allergic rhinitis and asthma, which may present *de novo* post-transplant or as exacerbations of pre-existing disease. Clinical manifestations typically include rhinorrhea, nasal congestion, sneezing, conjunctivitis, and, in the case of asthma, wheezing and dyspnea (4, 5). Beyond IgE-mediated disease, transplanted patients may also develop non-IgE-mediated immune disorders, such as eosinophilic gastrointestinal disorders, which include eosinophilic esophagitis, eosinophilic gastritis, gastroenteritis, enterocolitis, and colitis (6–9). The onset of food allergy after LT can range from days to years, depending on the presumed pathophysiological mechanism involved (10–17). When compared with adults, children are more likely to develop food allergy after LT, typically within 1–2 years (14, 18, 19). The most common clinical manifestations include angioedema, gastrointestinal symptoms such as vomiting and diarrhea, urticaria, eczema, and severe reactions such as anaphylaxis (14, 18, 20). Several studies investigated the risk factors associated with dnPTFA in pediatric population. Key contributors include the recipient's young age and female sex, familial and donor history of atopy, immunosuppressive therapy, and viral infection, particularly Epstein–Barr virus (EBV) and cytomegalovirus (CMV) (5, 14, 17). Tacrolimus, the most commonly used immunosuppressant in pediatric liver transplant recipients, inhibits calcineurin-dependent T-cell activation by reducing IL-2 transcription. However, its effects are not uniform across all T-cell subsets, and incomplete modulation of immune responses may contribute to a relative predominance of Th2 and Th17 pathways. In addition, tacrolimus has been associated with increased intestinal permeability, which may facilitate allergen sensitization (21, 22). EBV infection is another relevant factor associated with dnPTFA and may further contribute to immune dysregulation in this setting. EBV primarily infects B lymphocytes and can alter immune homeostasis by promoting an unbalanced Th2 response and compromising normal immune tolerance mechanisms, likely facilitating the development of allergic sensitization (3). Currently, the gold standard treatment for food allergies is strict elimination of the offending allergens from the diet; this paradigm also applies to patients who develop dnPTFA (18). Since 2024, Omalizumab—a monoclonal antibody—has emerged as a valuable option for pediatric patients with IgE-mediated food allergies. It reduces the risk of reactions from accidental exposures since it increases the threshold for clinical reactivity to common food allergens (23, 24).

## Case presentation

We report the case of a 15-year-old boy who underwent liver transplant at 28 days of age for a diagnosis of neonatal acute liver failure due to gestational alloimmune liver disease. He received a hyper-reduced left lateral segment liver graft from a deceased donor. The donor's cause of death was not related to allergic or anaphylactic events. Information regarding the donor's atopic history was not available; however, no allergic conditions were reported at the time of donation. The recipient had no family history of atopy. Post-transplant immunosuppression included induction with two basiliximab infusions (day 0–4) and three intravenous boluses of methylprednisolone (10 mg/kg at day 0–1–2). Thereafter, the patient was maintained on tacrolimus monotherapy, with progressive tapering to achieve therapeutic levels without nephrotoxicity or neurotoxicity (range between 3 and 5 ng/mL). The early post-transplant course was uneventful except for failure to thrive, with both length and weight under the third centile. Caloric intake was guaranteed through enteral nutrition via nasogastric tube with breast milk and the addition of extensively hydrolyzed formula. Two months after LT, he was discharged from the hospital. At regular follow-up visits, laboratory tests showed liver graft function consistently within limits, with protocol liver biopsies (at 6 months, 1 year, 2 years) negative for signs of acute or chronic rejection. TAC serum levels remained consistently within our center-defined target range (2–3 ng/mL beyond the first post-transplant year). CMV surveillance was negative, while EBV surveillance showed a very low chronic viremia [Log10 = 2 IU/mL] over time without any clinical symptoms. Medication adherence was excellent. At 6 months of age, the patient experienced his first episode of anaphylaxis after ingesting first cow's milk protein formula, presenting with acute dyspnea, angioedema, and urticaria. Subsequently, he presented with wheezing after inhalation exposure to hen's egg. Allergy testing revealed the following: total IgE 15,270 kU/L, cow's milk > 100 kU/L, bos d4 78.4 kU/L, bos d5 > 100 kU/L, bos d8 > 100 kU/L, egg white-specific IgE > 100 kU/L, gal d2 > 100 kU/L; ISAC test positive for cashew, hazelnut, soy, peanut, gliadin, peach, LTP, latex (Hev b1, Hev b3), ovalbumin, and milk fractions; skin prick test reactivity to cow's milk 9 mm, casein 6 mm, Betalattoglobulin 9 mm, Alfalattoalbumin 3 mm, and egg white 6 mm with egg yolk negative; and prick-by-prick positivity to donkey milk 3 mm and rice milk formula 9 mm. An elimination diet for milk and eggs was prescribed, with intramuscular epinephrine auto-injector. At 3 years, he underwent an oral provocation test with baked egg, which was tolerated. In the same year, the patient reacted to raw egg during a double-blind placebo-controlled food challenge. He had also experienced mild-to-moderate atopic dermatitis from the first month of life, with complete resolution at 4 years of age. His weight remained around the 3rd percentile, although height gradually normalized. Between 4 and 7 years of age, he experienced several allergic reactions, including five anaphylactic reactions, after accidentally eating baked and fresh milk. At 6 years, he developed anaphylaxis after consuming prepackaged pesto sauce. Due to this patient's adverse reaction and the uncertain composition of the pesto consumed, tree nuts were subsequently eliminated from his diet. At 9 years of age, oral food challenges (OFCs) for peanut, walnut, and hazelnut were negative, and these were then allowed in his diet. However, he experienced another episode of anaphylaxis when he was 10 years old after pistachio ingestion. At the same age, oral food challenge (OFC) with 4 mL fresh milk elicited wheezing, urticaria, and vomiting. The patient’s case was complicated by chronic respiratory symptoms. From 2 years of age, he required inhaled fluticasone propionate as maintenance therapy for recurrent wheezing. From 5 years of age, the patient required inhaled fluticasone propionate as maintenance therapy for recurrent wheezing. Because of frequent wheezing, chronic cough, and difficulty in performing everyday activities, he underwent baseline spirometry, with bronchodilator reversibility testing showing an obstructive pattern with FEV1 1.08L (66% of predicted) prebronchodilator and FEV1 2.38L (87% of predicted, increase of 240 mL from baseline). He was diagnosed with severe persistent asthma (Table 1). Serial spirometry evaluations performed during follow-up showed progressive improvement in lung function, culminating in normalization at the latest assessment (Table 1).

**Table 1:** Spirometry test pre- and postomalizumab therapy.

Parameter	Preomalizumab 9 years old		Postomalizumab^a^ 13 years old	
	Prebronchodilator	Postbronchodilator	Prebronchodilator	Postbronchodilator
FEV1	1.08 L (66%)	1.32 L (87%) +240 mL	2.38 L (93%)	2.45 L (96%) +70 mL
FEV1/FVC	69%	86.9%	95%	95%
FVC	1.56 L (84%)	2.01 L (109%) +460 mL	2.51 L (84%)	2.58 L (86%) +70mL
Weight (kg)	22 (10th percentile)		45 (50th percentile)	
Height (cm)	131 (25th percentile)		163 (50th percentile)	

a After 525 mg subcutaneous omalizumab every 14 days.

The inhalant allergen prick test was positive for Dermatophagoides pteronyssinus, Dermatophagoides farinae, Alternaria, a mix of Gramineae, and olive. These exams supported the diagnosis of IgE-mediated asthma. Considering the respiratory morbidity of the patient and recurrent anaphylactic reactions that led to a markedly restricted diet with persistent weight at the third percentile, a dedicated therapeutic strategy was required.

Modification of the immunosuppressive regimen, including conversion to cyclosporine or the addition of mycophenolate mofetil (MMF), was carefully considered. However, given the patient's stable graft function under low-dose tacrolimus monotherapy and the history of severe, life-threatening allergic reactions, a therapeutic strategy with a more rapid and predictable onset of action was prioritized. Therefore, no change in immunosuppression was pursued, and treatment with omalizumab was initiated.

As a rapid and reliable intervention was required, omalizumab—an anti-IgE monoclonal antibody approved for moderate-to-severe pediatric asthma—was rather initiated despite the lack of regulatory approval for pediatric food allergy at that time. With pretreatment total IgE levels around 1,500 kU/L, subcutaneous omalizumab was started at 10 years of age at a dose of 525 mg every 14 days.

**Figure 1:**
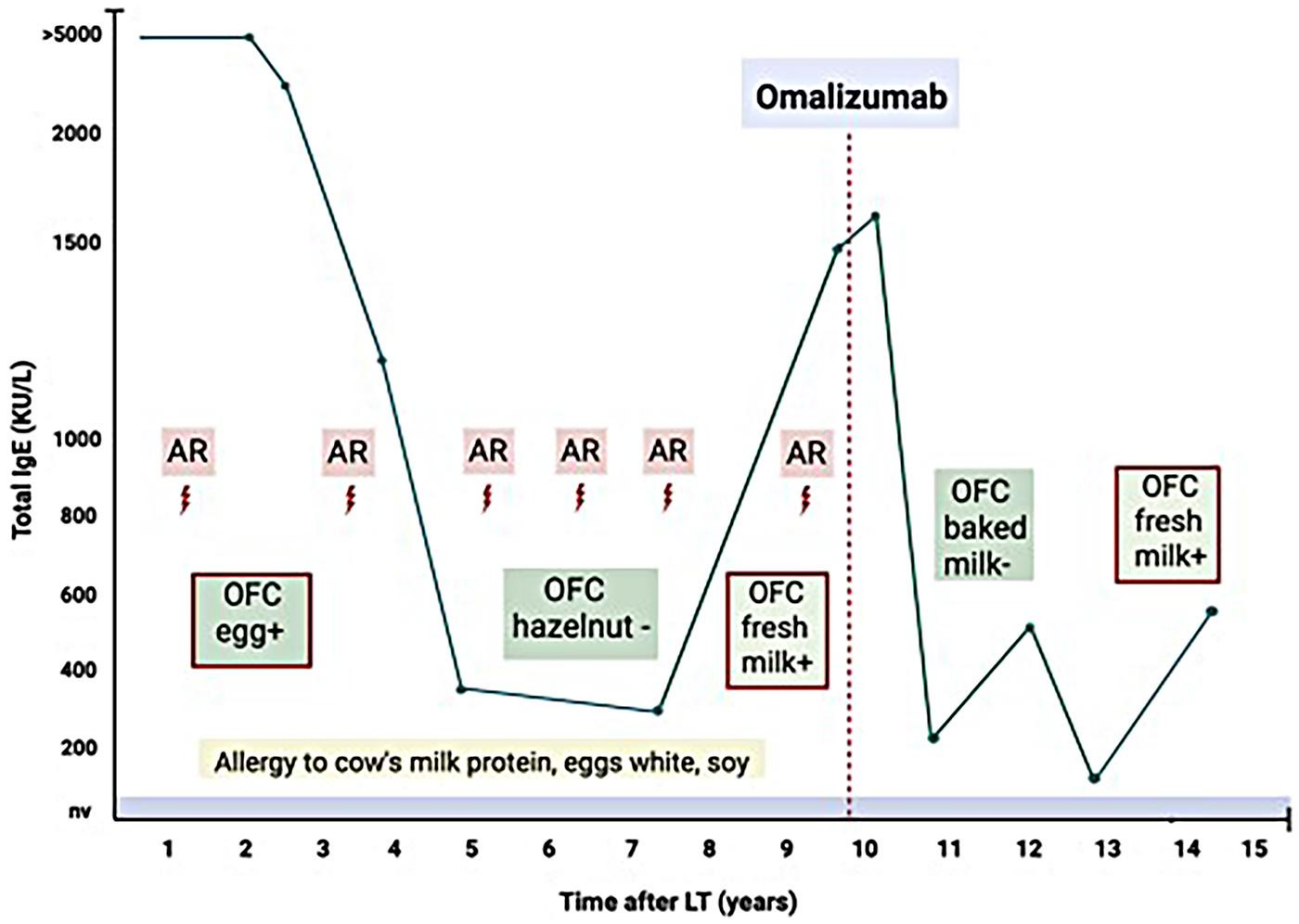
Total IgE levels and OFC before and after subcutaneous omalizumab therapy. Trends in total serum IgE levels over time in relation to oral challenge tests (OFCs) and the initiation of omalizumab therapy. IgE values show a progressive initial reduction, followed by variations after the introduction of treatment. The red bolts represent anaphylactic reaction (AR). The boxes indicate the OFC results: positive tests (+) are in red and negative tests (−) are in green, relating to different foods (egg, fresh milk, cooked milk, peanut, walnut, and hazelnut). The line represents total IgE levels (IU/mL) at the different time points; the numbers next to the points indicate the measured values. The initiation of omalizumab therapy is highlighted and coincides with a subsequent modulation of clinical and laboratory response. Figure created using BioRender.com (Toronto, Canada).

Following the initiation of biological therapy, exacerbation frequency and oral corticosteroid use significantly decreased. One year later, the patient underwent two OFCs: the first with baked milk, which was tolerated, and the second with fresh cow's milk, which elicited mild anaphylaxis. Total IgE levels showed an initial mild increase to 1,867 kU/L after 6 months, followed by a marked reduction after 9 months to levels under 650 kU/L, as illustrated in Figure 1. Spirometry levels improved, with FEV1 reaching 93% of the predicted value and FEV1/forced vital capacity (FVC) ratio 95%, alongside a negative bronchodilator reversibility test, as shown in Table 1. No further anaphylaxis and acute asthma episodes occurred. At the most recent evaluation, the patient demonstrated excellent catch-up growth, with height at the 50th percentile and weight at the 50th percentiles, and was able to reintroduce baked milk into his diet.

## Discussion

To the best of our knowledge, this case represents the first reported attempt to treat severe TAA with omalizumab in a pediatric LT recipient. In our patient, therapy proved to be safe and effective over a long-term follow-up of 5 years. Treatment resulted in excellent asthma control, elimination of anaphylactic events, and meaningful expansion of dietary tolerance, while maintaining normal graft function. TAA, particularly dnPTFA, is a recognized complication in pediatric patients after LT. A New Zealand study evaluating 60 long-term liver transplant survivors, most of whom had undergone transplantation in childhood, reported that 40% developed TAA and 36% developed dnPTFA, typically diagnosed within 1–4 years post-transplant. Notably, 10% of the latter group of patients had developed allergies severe enough to warrant the use of epinephrine auto-injectors (5). Similarly, Almaas et al. reported dnPTFA in 39% of 59 LT recipients, occurring between 6 months and 3 years post-transplant (25). In a Canadian study of 273 pediatric solid organ transplant recipients, authors found higher rates of TAA in multivisceral transplant recipients (57%) compared with LT (40.5%) and heart transplant (40%), with an overall dnPTFA prevalence after SOT of 25.3% (10). The increased risk of food allergies after solid organ transplantation parallels the increased risk of asthma after LT through shared mechanisms of Th2-mediated immune dysregulation and atopic progression (3). Almaas et al. demonstrated that transplanted children with asthma had 3.6-fold higher rates of sensitization to food allergens compared with those without asthma (RR 3.6, 95% CI 1.3–10.3) (25). In our case, the first episode of food allergy occurred 6 months after transplantation, consistent with the early onset documented in the literature, particularly in cases of severe presentation (20). International guidelines provide treatment protocols for the use of antihistamines and steroids in cases of an immediate allergic reaction to food and intramuscular epinephrine in cases of anaphylaxis (26). However, in recent years, biological therapies have emerged as potential maintenance strategies aimed at preventing reactions due to food allergy. Omalizumab acts by binding circulating IgE, preventing its interaction with high-affinity IgE receptors (FcεRI) on mast cells and basophils, leading to receptor downregulation. This results in reduced allergic inflammation and increased tolerance to allergens (24). Since 2003, omalizumab has been indicated for moderate-to-severe persistent asthma in patients aged 6 years and older with a positive skin test or *in vitro* reactivity to a perennial aeroallergen, the symptoms of which remain inadequately controlled despite inhaled corticosteroid therapy. In 2014, omalizumab was approved for chronic idiopathic or spontaneous urticaria in both the United States and Europe, particularly for patients who remain symptomatic despite H1-antihistamine treatment (26). The most significant addition was in February 2024 when the FDA approved omalizumab for IgE-mediated food allergy in certain adults and children aged 1 year and older (27). In a recent Italian study, Arasi et al. proved that omalizumab increased food allergen threshold in patients treated for severe asthma with food allergy comorbidity (28). Mori et al. proposed a new protocol for management of dnPTFA, which considered the use of biological drugs to treat severe food allergies; no contraindications have been identified for this population in the literature (18). In our patient, treatment with omalizumab gave us the opportunity to repeat the oral food challenge with cow's milk, which was tolerated in baked form but not fresh. Given the patient’s history of severe anaphylaxis, the increased tolerance threshold represented a clinically meaningful therapeutic achievement, allowing greater dietary diversification, reducing the risk of severe allergic reaction, and permitting discontinuation of epinephrine use. Several studies suggest that modifying the immunosuppressive regimen may enhance the effectiveness of dietary elimination and promote resolution of dnPTFA (19, 29–31), although the results are not consistent (17, 32). Haflidottir et al. suggest that adding MMF to TAC is associated with a reduced incidence of dnPTFA (12.5% vs. 37.8%, *P* = 0.003) compared with not adding MMF (33). In a systematic review conducted in 2022, the switch from TAC to Cys allowed the reintroduction of food allergens in 71.4% of patients (15/21) (34). Maarof et al. reported that 7/7 patients who developed food allergy (FA) after LT resolved after switching from TAC to Cys (35). However, most reports supporting conversion from tacrolimus to cyclosporine derive from relatively small and early case series, and the overall level of evidence remains limited. Moreover, because cyclosporine provides comparatively lower immunosuppressive potency, the potential immunologic benefit must be carefully balanced against the risk of graft rejection (32). More recently, due to their antiproliferative properties or the addition of mycophenolate, conversion from tacrolimus to mTOR inhibitors has also been associated with favorable outcomes in limited studies (17, 20, 31, 35). In our patient, modification of immunosuppression was not considered the most appropriate strategy. In light of his history of life-threatening allergic reactions, treatment with a rapid and predictable onset of action was prioritized over strategies based on immunosuppressive adjustment, the effects of which are typically slower and less predictable. Indeed, the use of omalizumab was off label for food allergy at that time, so dosing, duration, and safety could not have been standardized or compared with established protocols. To the best of our knowledge, this case study is the first reported in the literature about a pediatric patient who developed dnPTFA that was treated in the long term with omalizumab. We demonstrated its multisystem therapeutic efficacy, as treatment ensured excellent asthma control (allowing discontinuation of maintenance therapy), eliminated anaphylactic events, and enabled expansion of food tolerance. Omalizumab therapy also proved safe in this liver transplant recipient, improving quality of life without any side effects. Quality-of-life improvement was clinically assessed based on patient and caregiver reports. Moreover, this innovative therapeutic approach had a positive impact on the patient’s growth. A significant catch-up growth was observed after initiation of therapy, with height increasing from the 3rd to the 50th percentile and weight from the 25th to the 50th percentile.

Furthermore, it is important to recognize that factors such as advancing age and the natural development of immune tolerance may have contributed, at least in part, to the improvement observed in the manifestations of food allergy. However, in our case, anaphylaxis was frequent during the post-transplant years. Although the follow-up is substantial, this study does not provide systematic long-term outcome measures (e.g., standardized QoL scores, spirometry curves over time). The patient was regularly monitored through clinical evaluation and laboratory testing, including total IgE levels, although spirometry could not always be performed due to lack of patient compliance.

## Conclusion

This case report highlights the potential role of omalizumab as a long-term therapeutic option for severe IgE-mediated food allergy and concomitant severe asthma in pediatric liver transplant recipients. Further prospective studies with extended follow-up are required to identify the patient subgroups most likely to benefit and to define the optimal timing and clinical integration of biologic therapy in the management of severe dnPTFA.

## Data Availability

The raw data supporting the conclusions of this article will be made available by the authors, without undue reservation.
